# Circulating Tumor DNA correlates with Lactate Dehydrogenase, CYFRA 21-1, and CRP levels in patients with advanced NSCLC

**DOI:** 10.7150/jca.78574

**Published:** 2023-01-01

**Authors:** Marcela Buresova, Lucie Benesova, Marek Minarik, Renata Ptackova, Tereza Halkova, Petr Hosek, Jan Baxa, Milos Pesek, Martin Svaton, Ondrej Fiala

**Affiliations:** 1 Department of Pneumology and Phthisiology, Charles University, Faculty of Medicine in Pilsen, University Hospital in Pilsen, Pilsen, Czech Republic.; 2 Center for Applied Genomics of Solid Tumors, Genomac Research Institute, Drnovská 1112/60, Prague, Czech Republic.; 3 Elphogene, Drnovská 1112/60, Prague, Czech Republic.; 4 Department of Analytical Chemistry, Faculty of Science, Charles University, Hlavova 2030/8, Prague, Czech Republic.; 5 Laboratory of Cancer Treatment and Tissue Regeneration, Biomedical Center, Faculty of Medicine in Pilsen, Charles University, alej Svobody 76, Pilsen, Czech Republic.; 6 Department of Imaging Methods, Faculty of Medicine and University Hospital in Pilsen, Charles University, alej Svobody 80, Pilsen, Czech Republic.; 7 Department of Oncology and Radiotherapeutics, Faculty of Medicine and University Hospital in Pilsen, Charles University, alej Svobody 80, Pilsen, Czech Republic.

**Keywords:** NSCLC, LDH, CYFRA 21-1, ctDNA, prognosis

## Abstract

**Purpose:** To investigate potential association between selected tumor markers and laboratory parameters (lactate dehydrogenase [LDH], neutrophils, hemoglobin, neutrophils, lymphocytes, C-reactive protein, albumin, carcinoembryonic antigen, and cytokeratin 19 fragment 21-1 [CYFRA 21-1]) and circulating tumor DNA (ctDNA) with survival in patients with advanced non-small cell lung cancer (NSCLC).

**Patients and Methods:** The study encompassed 82 patients from a single center. All patients had (localy-) advanced adenocarcinomas. ctDNA was determined before starting therapy and at 6 weeks follow-up. Laboratory parameters were measured before each cycle of therapy and oncomarkers before starting the therapy as standard clinical practice. Mann-Whitney *U* test, Cox proportional hazards model, Fisher's exact test, and Kaplan-Meier survival estimation with Gehan-Wilcoxon test were used for statistical analysis of the corresponding variables.

**Results:** We have confirmed predictive or prognostic significance for some of the selected laboratory markers and oncomarkers. Above all, we demonstrate a significant relationship between the levels of LDH and the oncomarker CYFRA 21-1 and the presence or absence of ctDNA at the time of diagnosis. We also demonstrate significantly lower CRP levels in patients within whom the ctDNA disappeared during treatment. A similar but statistically insignificant trend was observed for LDH.

**Conclusions:** CYFRA 21-1, LDH and probably CRP correlate with ctDNA levels in NSCLC. Repeated measurement of these markers could thus help in early detection of disease progression in the same way as does ctDNA monitoring.

## Introduction

In spite of great therapeutic advances in treating non-small cell lung cancer (NSCLC), we still lack adequate prognostic and predictive markers for routine clinical practice in patients without driver mutations. Although programmed death-ligand 1 (PD-L1) expression may serve as a guide for immunotherapy, it is not an ideal marker for spatial and temporal heterogeneity 1. Moreover, PD-L1 does not determine the effectiveness of chemotherapy, which is a key part of treating patients with up to 50% PD-L1 expression and without targetable mutations 2.

Other NSCLC markers include inflammatory markers and lactate dehydrogenase (LDH) 3, 4. In previous publications, laboratory parameters of inflammation, including elevated C-reactive protein (CRP), higher neutrophils (Neu), lower albumin (Alb), and lower hemoglobin (Hb), appeared to be not only negative prognostic but also negative predictive markers for chemotherapy and immunotherapy effectivity 5-7. Similar results have been suggested for LDH 4, 6, 8. NSCLC has its specific oncomarkers, including cytokeratin 19 fragment (CYFRA 21-1) and carcinoembryonic antigen (CEA) for lung adenocarcinomas 9. These markers, too, possess prognostic and perhaps some predictive potential in NSCLC, as reported from previous studies mainly in patients treated with chemotherapy 10-12.

Another promising marker is circulating tumor DNA (ctDNA). ctDNA is used today in a method known as liquid biopsy. As this method is minimally invasive (based upon blood or other body fluid samples), it has appeal as a future cancer biomarker for diagnostics as well as for follow-up monitoring during systemic therapy 9, 11. In our previous work, we suggested ctDNA to be a promising prognostic and predictive marker for NSCLC patients receiving chemotherapy, which remains the cornerstone of stage IV treatment for most patients with NSCLC 13.

However, the relationship between the laboratory parameters or oncomarkers in NSCLC and ctDNA has not been evaluated extensively in prior studies. As mentioned above, ctDNA as well as laboratory markers or oncomarkers can have a prognostic or even predictive effect in patients with NSCLC. Therefore, we believe that the effort to learn the relationship between these parameters is beneficial for further progress in precision NSCLC oncology. The aim of our study, therefore, was to investigate the possible relationship of inflammatory parameters, LDH, and oncomarkers to ctDNA in advanced NSCLC patients along with their relationship to prognosis and prediction of treatment efficacy.

## Patients and Methods

### Study design and treatment

This single-center observational study enrolled patients with newly diagnosed cytologically or histologically confirmed locally-advanced (III) or metastatic (IV) stage NSCLC treated with first-line chemotherapy (platinum doublet +/- bevacizumab) between 8/2017 and 3/2021. All patients that met inclusion criteria and signed informed concerned were included in the study. Inclusion criteria were: inoperable lung adenocarcinoma stage III (not suitable for chemoradiotherapy) and stage IV, performance status 0 or 1, signed informed concerned and proven ctDNA in a blood sample.

Tumor tissue was assessed for the presence of tumor-specific somatic mutations using a preselected panel of the most commonly mutated genes in NSCLC (see below). All the patients were treated according to the current clinical guidelines using first-line chemotherapy regimens, including carboplatin plus paclitaxel plus bevacizumab, cisplatin or carboplatin plus pemetrexed, carboplatin plus docetaxel plus bevacizumab, or cisplatin plus vinorelbine. The chemotherapy was administered intravenously at the standard approved doses. Clinical follow-up controls including physical examination, plain chest X-ray, and routine laboratory tests were performed every 3 weeks. Computed tomography was carried out after two cycles of chemotherapy and then CT was repeated after 2-3 cycles. The objective tumor response was assessed using the Response Evaluation Criteria in Solid Tumors 1.1 (RECIST 1.1) in terms of complete remission, partial remission, stable disease, and progressive disease 14. Blood samples for the assessment of ctDNA were collected before the initiation of systemic therapy and 6 weeks after the first cycle of chemotherapy. All laboratory parameters and oncomarkers were determined at the time of tumor diagnosis (+/- 2 weeks) and additional CRP and LDH measurements were taken also at the time of administering the second treatment cycle (i.e., 6 weeks after the first cycle of chemotherapy).

Informed consent was obtained from all the participants. The study protocol and form of informed consent for participants were approved by the Ethical Committee of the Faculty of Medicine and University Hospital in Pilsen, Charles University on 13 June 2016 and complied with the International Ethical Guidelines for Biomedical Research, the Declaration of Helsinki, and local laws.

### Tumor DNA and plasma-based ctDNA extraction and mutation analyses

Tumor biopsy specimens obtained during bronchoscopy or transthoracic biopsy (cytological or formalin-fixed paraffin-embedded tissue samples) were tested for the presence of tumor-specific somatic mutations using a preselected panel of the most commonly mutated genes in NSCLC, including *KRAS*, *TP53*, *BRAF*, and *PIK3CA*. Tumor DNA was isolated from all available samples using the commercial column-based kit GenElute™ Mammalian Genomic DNA Miniprep Kit (Sigma Aldrich, St. Louis, MO, USA) according to the manufacturer's instructions for the respective tissue material. Mutation analysis based on PCR amplification of gene fragments followed by heteroduplex formation and their separation and detection by denaturing capillary electrophoresis (DCE) was performed as described previously 15-17. Selected tumor DNA without any detected mutation meeting the sufficient amount and concentration requirements was analyzed in more detail by next generation sequencing (NGS) using the ArcherDx VariantPlex Solid Tumor panel on the Illumina platform (ArcherDx, Boulder, CO, USA). Whole blood samples were collected into stabilization blood collection tubes (Carolina Biosystems, Prague, Czech Republic). The plasma fraction was obtained by a two-step centrifugation of the whole blood within 6-54 h after collection and then immediately frozen at -20˚C. The assessment of baseline ctDNA and ctDNA at 6 weeks follow-up was performed in patients with confirmed tumor-specific somatic mutation. CtDNA was extracted from plasma using the commercial column-based QIAamp Circulating Nucleic Acid Kit (Qiagen, Dusseldorf, Germany) according to the manufacturer's instructions. Mutations in plasmatic ctDNA were detected by the aforementioned PCR/DCE-based heteroduplex method (heteroduplex analysis after amplification of the mutated tumor-specific gene fragment). The peaks on the DCE electropherogram - homoduplex from wild-type DNA fragments (homoWT), homoduplex from mutated DNA fragments (homoMUT), and two heteroduplexes formed by one wild-type and one mutated DNA fragment (hetA and hetB) - were visualized by GeneMarker software (SoftGenetics, State College, PA, USA). Mutant allele frequency (MAF) was calculated according to the following equation:

(homoMUT+(hetA/2)+(hetB/2))/(homoWT+hetA+hetB) × 100)

The lowest MAF that could be detected and that was distinguishable from background or negative control (i.e., the limit of detection) was determined for each marker tested in plasma (about 0.1% depending upon the mutation being detected, data not shown). ctDNA clearance was defined as undetectable ctDNA levels in the plasma during the course of systemic therapy.

### Laboratory markers and oncomarkers

Laboratory parameters (CRP, Neu, lymphocytes [Lympho], Hb, Alb, LDH) and oncomarkers (CEA and CYFRA 21-1) were determined as a standard part of treatment in accredited laboratories of the University Hospital in Pilsen by certified methods commonly used in routine practice.

### Statistics

Standard frequencies and descriptive statistics were used to characterize the patient sample. Measured marker levels were compared between the two patient groups based on RECIST treatment response categories (CR+PR+SD vs. PD) using the Mann-Whitney *U* test. The same test was used to compare the time change of CRP and LDH between patients achieving and not achieving ctDNA clearance. For the survival analysis, progression-free survival (PFS) was determined from initiation of the therapy to the date of disease progression or death. Overall survival (OS) was determined from initiation of the therapy to the date of death. Patients who had not reached the PFS or OS endpoint were censored at the date of the last follow-up. The association of marker levels with OS and PFS was first explored in a continuous fashion using univariable Cox proportional hazards model. Subsequently, the patients were divided into two groups according to the clinical norms of each marker and PFS or OS in these groups were compared using the Kaplan-Meier method with the Gehan-Wilcoxon test and hazard ratio estimation by the Cox model. The relationship between marker levels and presence of ctDNA before the therapy was tested with Fisher's exact test using the same patient grouping according to the normal clinical levels.

All reported *p*-values are two-tailed and the level of statistical significance was set at *α* = 0.05. Statistical processing and testing were performed in STATISTICA (Version 12; StatSoft, Inc., Tulsa, OK, USA) and Matlab (2021a, MathWorks, Natick, MA, USA).

## Results

### Patient characteristics

In total, 107 patients signed informed concern, from that 82 patients met inclusion criteria and were evaluated to this study. The analyzed dataset consisted of 67.1% men. Median age was 66 years. Patient characteristics are summarized in **Table 1**.

### Association between laboratory parameters or oncomarkers and treatment response

We observed no significant relationship between the laboratory parameters or oncomarkers and the response to chemotherapy. Results are summarized in **Table 2**.

### Associations between laboratory parameters or oncomarkers and PFS or OS

In a continuous analysis using the univariable Cox proportional hazards model, we observed significantly poorer PFS for higher Neu (hazard rate = HR = 1.114, 95% confidence interval = 95% CI = 1.003-1.237, *p* = 0.043) and higher CYFRA 21-1 (HR = 1.022, 95% CI = 1.002-1.042, *p* = 0.027) and poorer OS for higher CRP (HR = 1.006, 95% CI = 1.001-1.010, *p* = 0.024) and CYFRA 21-1 (HR = 1.020, 95% CI = 1.003-1.038, *p* = 0.019) and better OS for higher albumin (HR = 0.908, 95% CI = 0.855-0.964, *p* = 0.002). All results are summarized in **Table 3**.

**Figure 1:**
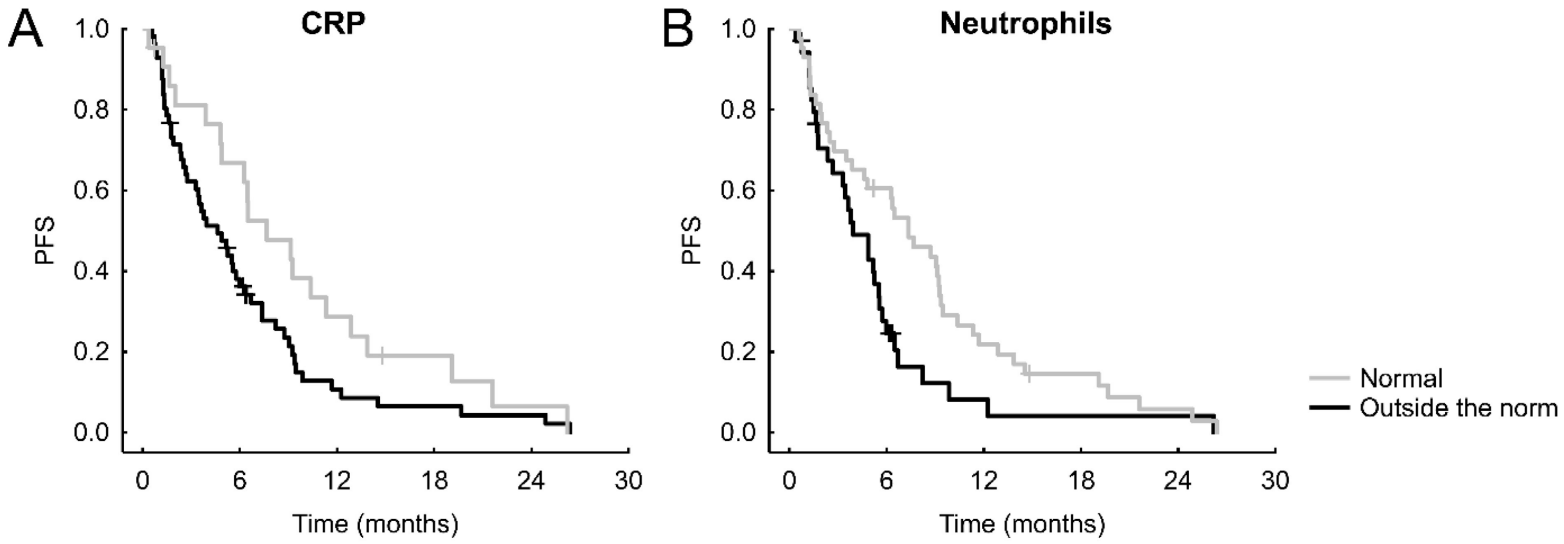
Significant Kaplan-Meier curves of laboratory markers and oncomarkers for PFS.

**Figure 2:**
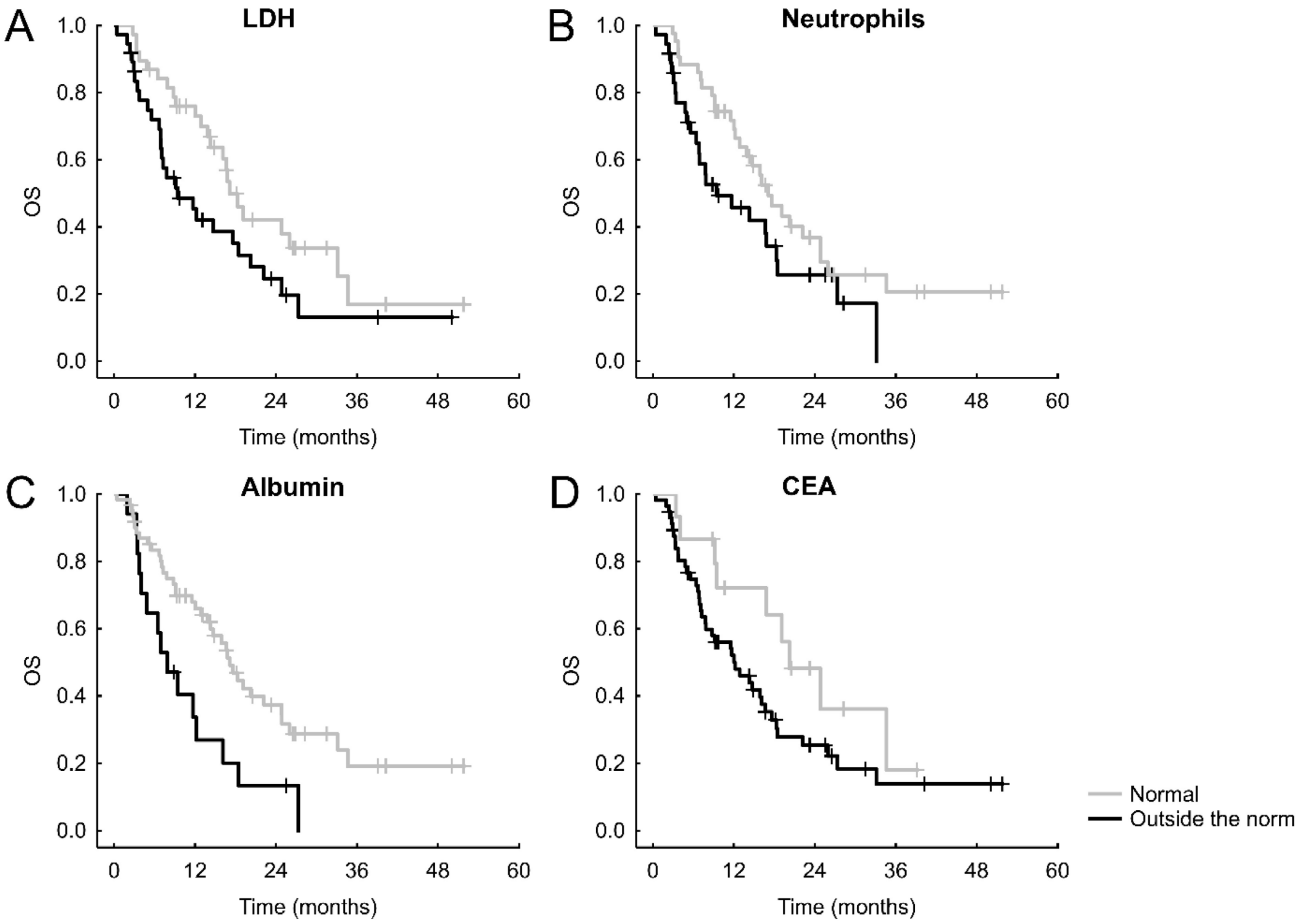
Significant Kaplan-Meier curves of laboratory markers and oncomarkers for OS.

After stratifying the patients according to normal or abnormal values of the parameters or oncomarkers, we recorded the following significant results: normal CRP was connected with better PFS (hazard ratio = HRo =1.629, 95% CI = 0.963-2.753, *p* = 0.036, **Figure 1A**), which was the case also for an unraised neutrophil count (HRo = 1.770, 95% CI = 1.085-2.888, *p* = 0.027, **Figure 1B**). Shorter OS was significantly connected with albumin lower than norm (HRo = 2.256, 95% CI = 1.227-4.145, *p* = 0.019, **Figure 2A**), increased neutrophils (HRo = 1.663, 95% CI = 0.962-2.873, *p* = 0.027, **Figure 2B**), high LDH (HRo = 1.696, 95% CI = 0.970-2.966, *p* = 0.024 **Figure 2C**), and elevated CEA (HRo = 1.796, 95% CI = 0.869-3.710, *p* = 0.048, **Figure 2D**).

### Association between laboratory parameters or oncomarkers and ctDNA

We observed a statistically significant association between higher LDH and ctDNA detection (*p* = 0.003) and between higher CYFRA 21-1 and ctDNA detection (*p* = 0.0004). Results are summarized in **Table 4**.

### Association between CRP or LDH change and ctDNA change

**Figure 3:**
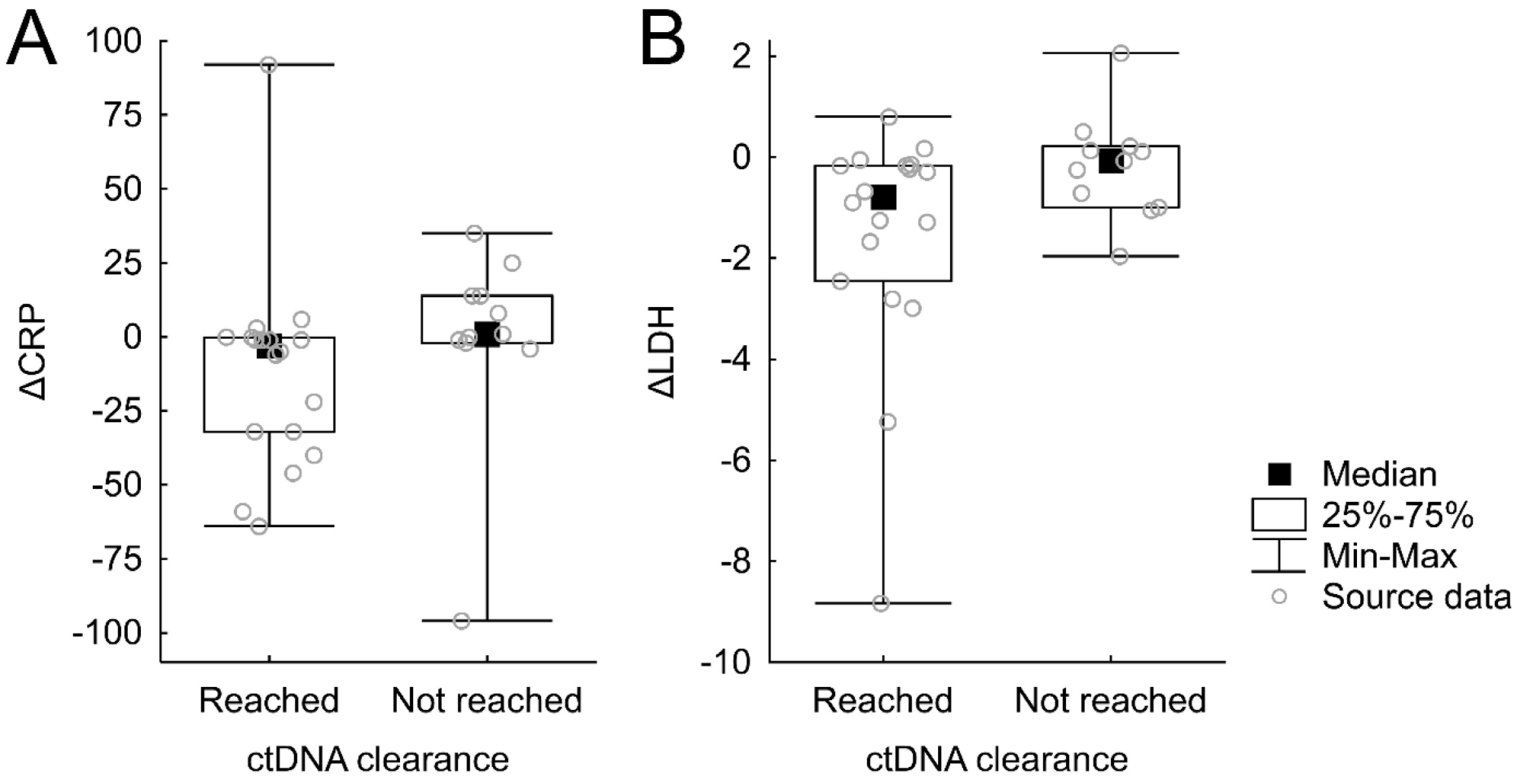
Relationships between CRP and LDH changes and ctDNA change.

CtDNA clearance after 6 weeks was significantly associated with a reduction of CRP (*p* = 0.047, **Figure 3A**). For LDH, a similar but statistically nonsignificant trend was observed (*p* = 0.069, **Figure 3B**).

## Discussion

As already mentioned, both ctDNA and laboratory markers or oncomarkers may influence the prognosis or prediction of NSCLC patients. Our findings uniquely point out correlation between ctDNA and CYFRA 21-1 and LDH in metastatic NSCLC. The relationships between these parameters have been only little researched so far. To the best of our knowledge, the relationship of ctDNA to laboratory markers or oncomarkers in NSCLC has not been investigated as extensively as in our study. We therefore believe that the results obtained by us will provide a topic for their better understanding.

The influence of certain laboratory parameters of inflammation or oncomarkers on the prognosis and/or prediction of treatment in patients with NSCLC that we have demonstrated here are in line with our previously published results. We demonstrated similar data across different types of treatment of advanced NSCLC patients, including chemotherapy, chemotherapy combined with anti-angiogenic therapy, and treatment with erlotinib 5-8, 18, 19. The effect we demonstrated here (taken as continuous or categorical variables) for CRP, Neu, Alb, CEA, and CYFRA 21-1 in relation to PFS and/or OS only highlights this issue in our group of patients treated with first-line chemotherapy for advanced NSCLC. We did not demonstrate an influence of any of these parameters on the objective response to treatment. That might be due to the relatively low number of patients.

The main aim of this study was to investigate possible association of these parameters with ctDNA. Several previous studies have addressed a similar relationship between LDH and circulating free DNA (cfDNA) in NSCLC 20, 21. Similar to our study performed only on the NSCLC-derived cfDNA fraction (i.e., ctDNA), higher levels of cfDNA correlated with higher level of LDH. It is known that cfDNA can also originate from benign processes 21, and it is even described that cfDNA from processes other than cancer (e.g., systemic inflammation, trauma) can make up the majority of cfDNA 22. For this reason, the specificity of cfDNA may not be high, and from our point of view direct determination of ctDNA seems to be more appropriate.

Several studies with other types of tumors point to relationships between ctDNA and laboratory parameters. Forthun et al. and Lee et al. described an association between higher LDH and ctDNA levels in patients with malignant melanoma 23, 24. A similar relationship between LDH and ctDNA also has been described for Hodgkin's lymphoma and colorectal cancer 25, 26. However, the relationship between laboratory parameters and ctDNA in NSCLC had not been investigated until a recent study by Low et al. brought the first results on this topic 27. Using a similar number of patients as did we (n = 79), they investigated the relationship between ctDNA and LDH, CRP, or white blood cells (WBC). For WBC, they did not observe a significant relationship to ctDNA (although they did not distinguish WBC subtypes), and that was consistent with our results for Neu and Lympho. As in our study, they similarly found a relationship between higher LDH and ctDNA levels. In contrast to our results, they also found a significant relationship between high CRP and ctDNA levels. This could be due to the representation of other histological types of NSCLC and to a different detection method used for ctDNA (based on large NGS panel) that reveals a wider spectrum of mutations, but, in comparison to our method, may be less sensitive to the detection of ctDNA in individual patients. On the other hand, we noted significant changes in ctDNA correlated with the change in CRP. In our opinion, the influence of changes in ctDNA on the CRP level deserves further research. Overall, the relationship between LDH and ctDNA could be explained by the hypothesis expressed in a publication by Kumar et al. suggesting that, because LDH is a marker of tissue breakdown, LDH may reflect a higher turnover rate of tumor with a greater occurrence of cell death and therefore a greater production of ctDNA 21.

The possible relationship between ctDNA and selected oncomarkers has already been investigated in smaller studies in tumors other than NSCLC, albeit with conflicting results 28-30. Although, for example, a relationship between CEA and ctDNA was described in colorectal cancer, no relationship to CEA was observed in gastric cancer 29, 30. Similarly, in pancreatic cancer, no relationship between either CEA or CYFRA 21-1 and ctDNA was observed 28. Therefore, in our study, we sought to clarify any possible relationship between CEA and/or CYFRA 21-1 and ctDNA in NSCLC. We observed a significant relationship between CYFRA 21-1 and ctDNA but not between CEA and ctDNA. We have identified only a few studies addressing similar correlations in NSCLC. Dietz et al. present the case report of a patient harboring anaplastic lymphoma kinase (ALK) translocation in whom ctDNA, CEA, and CYFRA 21-1 levels all increased during progression 31. Zhang et al. then investigated the effect of ctDNA changes on CEA 32. Their study included only 14 patients (just 7 of which were adenocarcinomas), however, and therefore, although they observed a certain trend in the relationship between elevated CEA and ctDNA, their results did not reach statistical significance. The largest study we have found is the aforementioned study by Low et al., who describe the correlation between ctDNA positivity and CEA 27. In their study, they found ctDNA in 79 patients, the majority of whom had adenocarcinomas. They used a broader NGS panel that also included other types of mutations. A finding that is in contrast to our study, which found that CEA level did not correlate with ctDNA positivity, can thus be explained by that relationship's being linked only to the specific mutational spectrum. For future studies, it would therefore be appropriate to assess these correlations also with regard to individual types of mutations.

Limitations of our study include the low number of patients and the fact that the study uses retrospective data of laboratory parameters and oncomarkers. For this reason, the values of all markers for each of the patients were not known before the start of therapy, and, at the same time, we could not evaluate the influence of the dynamics of oncomarkers, because we do not have repeated samplings available for oncomarkers. We also cannot fully exclude the influence of other benign diseases on the levels of laboratory markers and oncomarkers. Last but not least, our set of patients was not sufficient for the external validation of the results we obtained. However, we understand this study as exploratory in this respect, with an effort to bring new possibilities for further research. For these reasons, a prospective validation of our data on a larger group of patients would be appropriate.

## Conclusions

In our study, we confirm possible prognostic and/or predictive influence of selected laboratory parameters and oncomarkers in patients with advanced NSCLC treated with first-line chemotherapy. In particular, we point out correlation between levels of LDH and CYFRA 21-1 and ctDNA before the start of therapy. This relationship is also evidenced by the significant reduction of CRP when ctDNA disappears during treatment. We observed the same trend for LDH.

## Figures and Tables

**Table 1:** Patient characteristics

Parameter	Category	n (%)
Gender	Male	55 (67.1)
	Female	27 (32.9)
Smoking	Non-smoker	11 (13.4)
	Ex-smoker	22 (26.8)
	Smoker	49 (59.8)
TNM stage at diagnosis (simplified)	T1	4 (5.0)
	T2	18 (22.5)
	T3	15 (18.8)
	T4	43 (53.8)
	TX	2 (-)
	N0	6 (7.3)
	N1	11 (13.4)
	N2	28 (34.1)
	N3	37 (45.1)
	M0	11 (13.4)
	M1	71 (86.6)
Stage	IIIA	3 (3.7)
	IIIB	4 (4.9)
	IIIC	4 (4.9)
	IVA	21 (25.6)
	IVB	49 (59.8)
	IVC	1 (1.2)
RECIST at 1^st^ restage	CR	1 (1.4)
	PR	18 (24.7)
	SD	37 (50.7)
	PD	17 (23.3)
	Unknown	9 (-)
ECOG PS	0	1 (1.2)
	1	68 (82.9)
	2	13 (15.9)

RECIST: response evaluation criteria in solid tumors, CR: complete response, PR: partial response, SD: stable disease, PD: progressive disease. TNM classification in accordance with the 8^th^ edition.

**Table 2:** Relationship of laboratory markers and oncomarkers to treatment response

Marker (before starting treatment)	CR/PR/SD, median (25%-75%)	PD, median (25%-75%)	*p*-value
CRP (mg/l)	11.5 (3.6-56.0)	14.0 (6.0-62.0)	0.5824
LDH (µkat/l)	3.9 (3.2-4.9)	3.8 (3.3-5.1)	0.8409
Hb (g/l)	141 (129-151)	139 (133-146)	0.8513
Neu (10^9^/l)	6.6 (4.2-7.7)	6.8 (4.8-7.7)	0.7318
Lympho (10^9^/l)	2.1 (1.5-2.4)	1.8 (1.3-2.2)	0.2964
Alb (g/l)	40.1 (37.5-44.2)	40.7 (38.6-42.4)	0.9023
CEA (μg/l)	11.5 (3.3-30.1)	7.0 (3.8-26.9)	0.6300
CYFRA (μg/l)	3.6 (2.0-8.9)	3.8 (1.9-6.3)	0.8184

CR: complete response, PR: partial response, SD: stable disease, PD: progressive disease, CRP: C-reactive protein, LDH: lactate dehydrogenase, Hb: hemoglobin, Neu: neutrophils, Lympho: lymphocytes, Alb: albumin, CEA: carcinoembryonic antigen, CYFRA: cytokeratin 19 fragment.

**Table 3:** Relationship of laboratory markers and oncomarkers to PFS and OS

Marker (before starting treatment)	PFS		OS	
	*p*-value	Hazard rate (95% CI)	*p*-value	Hazard rate (95% CI)
CRP (mg/l)	0.293	1.003 (0.998-1.007)	**0.024**	**1.006 (1.001-1.010)**
LDH (µkat/l)	0.229	0.942 (0.855-1.038)	0.871	1.008 (0.921-1.103)
Hb (g/l)	0.400	0.994 (0.980-1.008)	0.335	0.992 (0.976-1.008)
Neu (10^9^/l)	**0.043**	**1.114 (1.003-1.237)**	0.079	1.090 (0.990-1.199)
Lympho (10^9^/l)	0.489	0.860 (0.560-1.319)	0.242	0.749 (0.462-1.215)
Alb (g/l)	0.414	1.003 (0.996-1.009)	**0.002**	**0.908 (0.855-0.964)**
CEA (μg/l)	0.104	1.001 (1.000-1.003)	0.209	1.001 (0.999-1.003)
CYFRA (μg/l)	**0.027**	**1.022 (1.002-1.042)**	**0.019**	**1.020 (1.003-1.038)**

PFS: progression-free survival, OS: overall survival, CRP: C-reactive protein, LDH: lactate dehydrogenase, Hb: hemoglobin, Neu: neutrophils, Lympho: lymphocytes, Alb: albumin, CEA: carcinoembryonic antigen, CYFRA: cytokeratin 19 fragment.

**Table 4:** Relationship of laboratory markers and oncomarkers to ctDNA

Marker (before starting treatment)		ctDNA present (control), n	ctDNA absent (control), n	*p*-value (Fisher's exact)
CRP (mg/l)	≤5	8	10	0.1517
	>5	28	14	
LDH (µkat/l)	≤4	12	17	**0.0033**
	>4	24	6	
Hb (g/l)	≥120F, 135M	25	22	0.0563
	<120M, 135F	11	2	
Neu (10^9^/l)	≤7	19	15	0.5960
	>7	17	9	
Lympho (10^9^/l)	≥0.8	36	24	-
	<0.8	0	0	
Alb (g/l)	≥37	25	20	0.3618
	<37	11	4	
CEA (μg/l)	≤3	7	2	0.2892
	>3	26	20	
CYFRA (μg/l)	≤2.5	5	14	**0.0004**
	>2.5	28	8	

ctDNA: circulating tumor DNA, CRP: C-reactive protein, LDH: lactate dehydrogenase, Hb: hemoglobin, Neu: neutrophils, Lympho: lymphocytes, Alb: albumin, CEA: carcinoembryonic antigen, CYFRA: cytokeratin 19 fragment, M: male, F: female.

## Abbreviations

Alb: Albumin
ALK: anaplastic lymphoma kinase
BRAF: a human gene encoding a protein called B-Raf
CEA: carcinoembryonic antigen
CRP: C-reaction protein
ctDNA: circulating tumor DNA
CYFRA 21-1: cytokeratin fragments
DCE: dynamic contrast enhanced
Hb: hemoglobin
KRAS: Kirsten rat sarcoma virus oncogene homolog
LDH: lactate dehydrogenase
Lympho: lymphocytes
MAF: minor allele frequency
Neu: neutrophils
NGS: next generation sequencing
NSCLC: non-small cell lung cancer
OS: overall survival
PD-L1: programmed death ligand 1
PIK3CA: phosphatidylinositol-4,5-bisphosphate 3-kinase catalytic subunit alpha
PFS: progression-free survival
RECIST: response evaluation criteria in solid tumors
P53: protein made by the TP53 gene
